# Identifying clinical error patterns in nursing students’ CPR performance: a mixed-methods OSCE study

**DOI:** 10.1016/j.resplu.2025.101089

**Published:** 2025-09-05

**Authors:** Kheizaran Miri, Alireza Sardashti, Sajedeh Moradi, Arefeh Naseri, Amirhossein Mirzaei

**Affiliations:** a Ph.D. MSc, BSc, RN, Department of Nursing, School of Nursing and Midwifery, Torbat Heydariyeh University of Medical Sciences, Torbat Heydariyeh, Iran; b Master’s degree student in Nursing, BSc, Student Research Committee, Torbat Heydariyeh University of Medical Sciences, Torbat Heydariyeh, Iran; c Undergraduate Nursing Student, Student Research Committee, Torbat Heydariyeh University of Medical Sciences, Torbat Heydariyeh, Iran

**Keywords:** CPR education, OSCE assessment, Clinical competence, Simulation training, Nursing errors, basic life support(BLS)

## Abstract

**Background:**

Basic Life Support (BLS) competency is critical for healthcare professionals, yet persistent performance gaps exist in training settings. This study aimed to identify error patterns in nursing students’ cardiopulmonary resuscitation (CPR) performance during Objective Structured Clinical Examinations (OSCEs) and, through stakeholder insights, identify perceived challenges and suggest remediation strategies to improve learning outcomes.

**Methods:**

A sequential explanatory mixed-methods study was conducted (2021–2024) with 250 eighth-semester nursing students. The quantitative phase analyzed OSCE performance using: 1) a validated 20-item BLS knowledge test, 2) a 17-item skills checklist, and 3) Laerdal QCPR® mannequin metrics. The qualitative phase included interviews with 25 stakeholders (students, instructors, examiners). Data were analyzed using descriptive/inferential statistics (SPSS v25) and thematic analysis (MAXQDA 2022).

**Results:**

Significant deficiencies were identified: 68 % failed to achieve guideline-compliant compression depth (mean: 40–50 mm vs. recommended 50–60 mm), 45 % demonstrated incorrect rates, and only 18 % properly applied AED pads. Qualitative analysis revealed stress (89 % prevalence) and inadequate feedback (75 %) as key performance barriers. Gender disparities emerged, with female students delivering 40 % shallower compressions (*p* < 0.05).

**Conclusion:**

This study reveals persistent gaps in nursing students’ ability to translate BLS knowledge into competent CPR performance during OSCEs. Deficiencies in compression quality, AED use, and decision-making highlight limitations of current training models. Integrating high-fidelity simulation, real-time feedback, and stress-mitigation strategies may strengthen skill acquisition. Further multicenter research is needed to establish evidence-based approaches that ensure graduates are prepared to deliver high-quality resuscitative care.

**Clinical trial number:** not applicable.

## Background

Basic Life Support (BLS) forms the foundation of effective cardiac arrest management, incorporating chest compressions, airway management, and the use of automated external defibrillators (AEDs). Competence in these skills is strongly linked to improved survival in emergencies.^1^ To evaluate such competence, healthcare education commonly uses Objective Structured Clinical Examinations (OSCEs) as standardized assessments of practical performance.^2^ However, despite structured training programs and clear international guidelines, recurring errors remain evident in both training and assessment. Frequent problems include delayed initiation of chest compressions or defibrillation, inadequate compression depth or rate, incomplete chest recoil, and improper airway maneuvers.^3^, ^4^, ^5^, ^6^ These errors often reflect not only technical deficiencies but also cognitive challenges such as stress-related decision-making or difficulty applying protocols under exam pressure. Multiple factors contribute to these shortcomings. Limited opportunities for repetitive practice, insufficient feedback, and the anxiety associated with OSCE settings can hinder skill demonstration.^7^ Moreover, the reliance on checklists for scoring has limitations: while useful for standardization, they may fail to capture the quality of cardiopulmonary resuscitation (CPR) performance. To address this, advanced evaluation methods—such as high-fidelity manikins and real-time feedback systems—are increasingly being employed, providing more objective and accurate assessments.^8^, ^9^, ^10^ Assessment reliability itself is another concern. Variability in examiner judgment and scoring rigor can influence results, raising the question of whether errors reflect genuine skill gaps or inconsistencies in exam administration.^11^ Mapping error patterns can therefore help clarify this distinction and support the development of more valid assessment tools. Although the literature widely describes BLS training and learner performance, relatively few studies have systematically examined specific error patterns within OSCE contexts or combined these findings with stakeholder perspectives. Bridging this gap is essential for designing interventions that directly address both observed performance issues and perceived barriers in training. Therefore, the present study aims to (1) identify and categorize error patterns in nursing students’ CPR performance during OSCE assessments, and (2) explore stakeholders’ views on challenges to effective BLS learning. By integrating these complementary perspectives, the study seeks to inform targeted improvements in training approaches that enhance both educational outcomes and future clinical performance.

## Methods

This longitudinal sequential explanatory mixed-methods study was conducted at Torbat Heydariyeh University of Medical Sciences from September 2021 to September 2024. The study consisted of two phases: quantitative and qualitative. This study adheres to the GRASPS (Guidelines for Reporting Simulation-Based Research in Healthcare Education) framework to ensure comprehensive reporting of simulation-based research elements.^12^ The GRASPS checklist has been followed to enhance methodological transparency, particularly in describing: (a) the high-fidelity simulation environment using Laerdal QCPR® technology, (b) standardized OSCE scenarios, and (c) objective performance metrics (supplementary file 1).

### Phase one: Quantitative research

This study employed a comprehensive quantitative approach examining 250 eighth-semester nursing students during their final OSCE assessments. Participant recruitment followed a standardized protocol: all eligible students (*N* = 265) received official university invitations, with 250 (94.3 %) providing written informed consent during registration. Explicit exclusion criteria were applied, including prior CPR certification (*n* = 12), incomplete OSCE participation (*n* = 3), or documented physical/psychological limitations (*n* = 0). Participants retained full withdrawal rights without academic consequences. The assessment protocol featured a rigorously designed 14-station OSCE administered across two days (8 min per station), with one dedicated BLS/CPR evaluation station. We implemented three validated instruments: (1) a demographic questionnaire (CVI = 0.92) capturing academic/clinical backgrounds; (2) a 20-item AHA-aligned BLS knowledge test (KR-20 = 0.75); and (3) a dual-assessment tool combining a 17-item cognitive checklist (KR-20 = 0.78) with practical evaluations using Laerdal QCPR® technology. This advanced system provided millimeter-precise measurements (±1 mm compression depth, ±2 compressions/min rate) through continuous data sampling aligned with AHA 2020 guidelines. The evaluation tool was developed according to *standard clinical resuscitation guidelines* (supplementary file 2) and builds upon the validated checklist used in prior studies, including Miri et al. (2024), which confirmed its content validity, construct validity, inter-rater reliability, and internal consistency.^13^ This instrument has been extensively employed in OSCE assessments within our institution. The tool systematically evaluates performance across four critical domains:

• **Chest Compressions** (depth, rate, recoil, and hand positioning)
• **Ventilation** (volume, rate, and technique)
• **Clinical Decision-Making** (timeliness and appropriateness of interventions)
• **AED Operation** (correct pad placement and shock delivery)

Within each domain, essential performance indicators were weighted according to their clinical significance. We subsequently calculated both error rates (procedural deviations) and success rates (correctly executed items) for each domain. This structured approach − combining domain-specific evaluation with weighted critical items − provides an objective, multidimensional assessment of resuscitation competency that aligns with contemporary simulation-based assessment standards.

### Phase two: Qualitative research

***Participant Recruitment and Selection***: The participant recruitment and selection process for this comprehensive qualitative study was designed to gather data through in-depth interviews with stakeholders. Through purposive sampling, we recruited 25 participants representing three key groups: 6 faculty members (recruited via departmental email invitations), 5 OSCE organizers (identified through institutional records), and 14 students (selected through stratified sampling based on their quantitative performance quartiles). All approached individuals agreed to participate, resulting in a 100 % response rate. Faculty and organizer participants were required to have a minimum of one year of OSCE experience to ensure relevant perspectives.

***Informed Consent and Ethical Considerations***: The study implemented rigorous consent procedures with two distinct protocols: written consent for individual interviews and audio-recorded verbal consent for focus group participation. Consent forms detailed study objectives, data confidentiality measures, voluntary participation rights, and audio recording permissions. All procedures received approval from the Institutional Review Board (IR.THUMS.REC. 402000038, with particular attention to voluntary participation, data anonymization, secure storage procedures, and participants' right to withdraw at any stage without consequences.

***Data Collection Procedures***: We conducted semi-structured interviews lasting 30–45 min that explored several critical dimensions: performance challenges in BLS stations, anxiety triggers during assessments, recurrent error patterns, training adequacy perceptions, and concrete suggestions for improvement. Examiner-specific interviews provided additional insights into frequency and patterns of technical errors, common mistake repetitions, and particularly challenging aspects of BLS execution. All interviews were transcribed verbatim within 72 h to ensure accuracy and immediacy of data processing.

***Data Analysis and Validation:*** The research team employed MAXQDA 2022 for thematic analysis of interview transcripts, supported by focus group discussions with 14 participants for validation. We implemented several strategies to ensure methodological rigor: expert panel validation (all CVIs > 0.8), four-month clinical immersion for contextual understanding, maintenance of reflexive researcher journals, and thick description of OSCE scenarios. Analytical integration was achieved through performance-quartile stratification and joint display analysis, enabling effective triangulation of quantitative and qualitative data.

***Quality Assurance and Reporting Standards:*** The study maintained the highest standards of qualitative research through prolonged engagement at the clinical skills center and meticulous documentation procedures. To ensure complete transparency and reproducibility, we have included a supplementary GRASPS checklist (Supplementary File 1) that details our compliance with simulation research standards, including specific protocols for participant briefing and debriefing procedures. This comprehensive approach allowed for systematic exploration of both objective performance metrics and subjective experiential factors in high-stakes BLS assessments.

## Result

The baseline characteristics of the participants are presented in Table 1. The mean age of the medical students was 21.8 ± 0.7 years, with an average GPA of 16.9 ± 0.8. Among the participants, 57.9 % were male and 42.1 % were female. More than half of the students (60.5 %) reported previous attendance in a CPR workshop, while 39.5 % had not participated in such training. Only 23.7 % indicated having prior work experience in a clinical setting, and 31.6 % had a history of CPR performance.

**Table 1:** Demographic variables of medical student in OSCE.

Variable	Frequency
Age (mean ± SD)	21.8 ± 0.7
GPA a(mean ± SD)	16.9 ± 0.8
Sex n (%)	
Male	(57.9)
Female	(42.1)
participated in CPR workshop^b^ n (%)	
Yes	(60.5)
No	(39.5)
work experience^c^ n (%)	
Yes	(23.7)
No	(76.3)
CPR experience n (%)	
Yes	(31.6)
No	(68.4)

a The grade point average (GPA) was calculated on a 0 to 20 grading scale.

b Participants who had attended a practical cardiopulmonary resuscitation (CPR) workshop within the last six months were excluded from the study. However, individuals who had only participated in theoretical or knowledge-based CPR workshops were included.

c The work experience refers to practical employment outside of required academic internships.

**Performance in Basic Life Support (BLS) Skills:** Detailed analysis of BLS skill performance is summarized in Table 2. Significant gaps were observed between guideline-based standards and actual student performance. Chest compressions showed the highest rate of error, with 68 % of participants failing to achieve the recommended depth of 50–60 mm, resulting in a success rate of only 32 %. Ventilation errors were also frequent, as 45 % delivered ventilations outside the correct rate (100–120 per minute). Incomplete chest recoil occurred in 29 % of students, while only 71 % achieved adequate recoil. Airway management was equally divided, with 50 % demonstrating proper technique. In contrast, some skills were performed more successfully. Correct tidal volume (500–600 mL) was achieved by 79 % of participants, and AED performance showed relatively high accuracy, with 82 % recognizing the device and 85 % applying pads appropriately. However, delays in initiating CPR/AED were reported in 32 % of cases, which may compromise timely intervention. All objective data regarding compression depth, rate, recoil, and ventilation volume were measured using the Laerdal QCPR® smart manikin, ensuring precise and standardized evaluation according to AHA guidelines.

**Table 2:** Comprehensive analysis of clinical errors and success rates in BLS skills.

**BLS Skill Category**	**Error Type**	**Standard Guideline**	**Error Frequency** **(%)**	**Success** **Rate** **(%)**	**Actual Value** **(Median ± IQR)**
**Chest Compressions**	Inadequate Depth*	50–60 mm	68 %	32 %	53 mm (48–57)
**Ventilation**	Incorrect Rate*	100–120 per minute	45 %	55 %	112/min (102–119)
	Incomplete Recoil*	Full recoil (100 %)	29 %	71 %	98 % (96–99)
	Airway Management	Proper technique	50 %	50 %	−
**Clinical Decision-Making**	Incorrect Volume*	500–600 mL	21 %	79 %	540 mL (490–590)
	Scene/Pulse Check	Assesses safety/checks pulse	32 %	68 %	**−**
**AED Operation**	CPR/AED Delay	Immediate action	32 %	68 %	**−**
	AED Recognition	Recognizes the device	18 %	82 %	**−**
	Improper Use	Correct pad placement	15 %	85 %	

* All objective measurements of CPR skills (including chest compression depth, compression rate, complete chest wall recoil, and ventilation volume) were performed using the Laerdal QCPR® smart manikin. This system provided high-precision measurements (±1 mm for compression depth and ±2 compressions per minute for rate) with continuous data sampling, enabling quantitative recording of errors based on AHA guidelines. The raw data were analyzed and reported by the manikin's dedicated software (QCPR Feedback).

**Challenges and Training Gaps in CPR Performance:** The most common challenges, procedural barriers, and suggested strategies are provided in Table 3. Several individual and contextual factors contributed to performance errors. Chest compression difficulties were often associated with fatigue, incorrect technique, and gender-related disparities in physical strength. Stress during OSCEs, limited manikin-based practice, and insufficient feedback were reported as training gaps that negatively affected skill retention. Ventilation errors were primarily attributed to tidal volume inaccuracies, airway mismanagement, and anatomical limitations. In AED operation, challenges included rhythm misidentification, forgotten procedural steps, and, in some cases, barriers related to color vision. Clinical decision-making errors commonly stemmed from delayed arrest recognition, prioritization difficulties, and legal or psychological concerns.

**Table 3:** Common challenges and recommended strategies in CPR performance.

**Performance Area**	**Individual /Procedural Challenges**	**Training/** **Evaluation Gaps**	**Recommended Strategies**
**Chest Compressions**	− Rapid fatigue (BMI/strength related) − Incorrect technique − Gender disparities	− Stress during OSCEs − Inadequate feedback − Limited manikin practice	1. Smart manikins with real-time feedback 2. Shorter, frequent practice sessions 3. BMI/gender-adjusted protocols
**Ventilation**	− Tidal volume errors − Poor airway management − Anatomical limitations − Poor airway management − Anatomical limitations	− Insufficient practice − Unrealistic simulations	1. Manikins with volume metrics 2. Repeated drills for mastery 3. Adaptive techniques for anatomy
**AED Operation**	− Rhythm misidentification^a^ − Forgotten steps − Color vision barriers	− Limited AED access − Over-reliance on theory	1. Hands-on AED practice 2. VR rhythm training 3. Color-blind friendly interfaces
**Clinical Decision-Making**	− Delayed arrest recognition − Prioritization errors − Legal fears	− Unpracticed scenarios − Ignored time pressure	1. Gradual scenario training 2. Case-based learning 3. Video review + cognitive training

a Rhythm confusion reflects self-reported uncertainty in identifying AED-appropriate rhythms, not measured performance.

To address these barriers, several strategies were recommended: the integration of smart manikins with real-time feedback, shorter but more frequent practice sessions, and adaptive protocols that consider individual differences (e.g., BMI, gender, or anatomical variations). Furthermore, scenario-based training, case-based learning, and the use of video reviews were emphasized as effective approaches for strengthening clinical decision-making under pressure.

## Discussion

This mixed-methods study provides a detailed examination of prevalent clinical error patterns during basic cardiopulmonary resuscitation (CPR) performance by nursing students in OSCE assessments. It proposes strategic interventions aimed at reducing these errors. This intervention should be based on our qualitative findings, preferably to remediate the mistakes of students. Our findings indicate that students suffer from a lack of proper feedback during the education period, which leads to the occurrence of stress during the OSCE. Subsequently, these problems cause students to demonstrate some errors during CPR performance, including improper compression depth and rate, not providing patient safety and required measurement in the golden time, and improper airway management. In the following paragraphs, we are going to discuss in detail how these background criteria will affect students' performance during OSCEs and try to provide some recommendations to improve this condition. The application of GRASPS guidelines in this study strengthens the validity of our findings by ensuring systematic reporting of all critical simulation components, from technical specifications of the QCPR manikin to the standardized administration of OSCE scenarios.

Students in our study declared that stress is an obstacle to achieving optimum scores in CPR stations. OSCE naturally is a main source of stress, even though raters are not very strict with students.^14^ But the OSCE is an instrument that has been invented to measure the ability of students to control their stress and show their skills that can comply with it, such as self-confidence.^15^ Stress is naturally happening since the sympathetic nervous system is becoming activated, leading to an increase in heart rate.^16^ However, stress is a must in stressful situations like this, because it increases attention and alertness.^17^ Furthermore, a moderate level of stress, regardless of whether it is high or low, can enhance creativity and lead to novel ideas.^18^ On the other hand, it is being claimed that stress impairs both cognitive and psychomotor skills^19^, ^20^. While in our study, it seems that psychomotor skills and decision-making were affected despite moderate knowledge. Therefore, it has been depicted in the study of Parvaneh Vasli et al. that achieving better academic scores leads to success in OSCE, but anxiety doesn’t have any role in it.^21^ It has been shown that excessive stress can impair cognition.^22^ Another challenge that existing literature consistently reports is discrepancies between cognitive understanding and the practical execution of CPR.^23^ Abbreviately, it seems that stress reduces information received from the environment, subsequently leading to weak psychomotor functions.^24^ Studies have shown that the quality of CPR performance often falls below the recommended standards. For example, in a clinical in-hospital study, compression rates were below 80 per minute in 36.9 % of measured intervals.^25^ In a simulation study, 47 % of participants failed to achieve the recommended compression depth (greater than 40 mm) from the very beginning of CPR.^26^ Furthermore, evidence indicates that 5.7 % of hospitalized cardiac arrest patients did not receive immediate CPR despite guideline recommendations, and delays of more than two minutes in initiating resuscitation were significantly associated with lower survival rates.^27^ Stress can impair decision-making skills; as it has been shown in our study, 32 % of participants failed to assess scene safety before initiating CPR, a critical step emphasized in BLS guidelines.^28^

It can be said that there is no comprehensive research that has investigated these variables simultaneously. For future research recommendations, all of the intervening variables and their effect on CPR performance through high-fidelity manikins can be explored.

To decrease the adverse effects of stress, there are several strategies; for instance, in our context, we are providing workshops for CPR to prepare students. 60.5 % of students passed the mentioned workshops, but results suggest that traditional training methods may be insufficient in fostering competency. On the contrary, researchers implemented a coping program for the reduction of this anxiety, and the results confirmed its effectiveness.^29^ In a different article in which very strict criteria were used to measure stress, including saliva and cortisol tests, results indicate anxiety doesn’t have any connection with OSCE score, but it is being suggested that other variables, like self-confidence, might have confounded other ones.^30^ Another way to decrease stress is through feedback. It has been declared by students that our research lacks proper feedback before the OSCE exam, leading to poor scores. There are other types of feedback, for instance, Students have a greater tendency to the written feedback than normal.^31^ While they can use an online system that is available everywhere.^32^ Audio tape feedback can be used for every student, but it takes a lot of time for the instructor.^33^ More importantly, OSCEs can be utilized to enhance students' practical skills.^34^ Parallel to this, Formative OSCEs force students to correct their mistakes.^35^

While our findings demonstrate the critical need for improved feedback mechanisms in CPR training, the choice of instructional technology must be evidence-based. Recent 2025 resuscitation guidelines have raised concerns about virtual reality (VR)-only training approaches due to insufficient evidence for skill transfer to clinical practice.^36^ Although VR provides valuable low-stress repetition and was particularly useful during COVID-19 restrictions, augmented reality (AR) solutions may offer superior training outcomes. AR's unique capacity to overlay digital guidance on physical manikins makes it particularly effective for correcting the biomechanical errors we observed (e.g., inadequate compression depth), while maintaining crucial physical practice elements.^37^ This aligns with our participants' expressed need for more realistic, feedback-rich training environments.

Notably, our study revealed a gender disparity, with female participants delivering compressions 40 % shallower than their male counterparts. This observation supports that rescuer's height and BMI also affect compression quality, with taller individuals performing better in standing positions, although this can lead to over-compression risks.^38^ These findings call for gender-sensitive training adaptations, such as modified manikins or strength-conditioning exercises, as suggested by recent works.^39^ Furthermore, students persist in entering communication into the OSCE.^40^ So Future researchers should investigate communication through CPR in the OSCE exam.

Our results corroborate global trends in BLS training deficiencies. This disparity underscores the need for frequent, competency-based refresher courses, as sporadic training leads to rapid skill decay.^41^ Based on the study findings, we propose a comprehensive multi-modal approach to improve BLS training effectiveness. First, enhanced simulation-based training incorporating real-time feedback manikins should be implemented to address prevalent compression depth and rate errors, as demonstrated by López-López et al. (2024).^42^, ^43^ Second, stress inoculation training through gradual exposure to high-pressure scenarios should be adopted to mitigate OSCE-related performance anxiety, following the evidence presented by Kim et al. (2024).^44^ Third, gender-adaptive protocols including adjusted manikin resistance settings and modified two-rescuer CPR strategies should be developed to accommodate biomechanical differences, building on the work of Rogers et al. (2024).^44^ Finally, the current high-stakes OSCE model should be supplemented with frequent low-stakes formative assessments to reinforce skills in a less stressful learning environment, as recommended by Cooper et al. (2024). This integrated approach addresses the key psychomotor, cognitive, and affective domain challenges identified in our study while aligning with current evidence-based practices in Nursing education.

### Limitations

This single-center study of nursing students has three key limitations. First, the artificial manikin-based OSCE assessments may not fully reflect real patient scenarios, and our findings require validation in clinical settings. Second, the exam-induced stress environment differs substantially from real resuscitation contexts in both triggers and consequences. Third, our technology recommendations precede the 2025 guideline updates. These factors suggest the need for multi-institutional studies with: (1) diverse healthcare learners, (2) longitudinal skill retention assessment, and (3) updated technology comparisons under new standards.

## Conclusion

This study demonstrates that despite structured training and OSCE-based assessment, nursing students continue to face persistent challenges in applying basic life support (BLS) skills effectively. Errors in chest compression quality and AED application, compounded by stress and limited feedback, indicate a disconnect between theoretical instruction and clinical performance.

By integrating error pattern analysis with stakeholder perspectives, the study highlights the urgent need to refine current training models. Approaches that combine high-fidelity simulation, real-time feedback, and strategies to reduce performance anxiety hold promise for bridging the gap between knowledge and practice. Future multicenter and longitudinal research should test these interventions systematically to establish evidence-based improvements in resuscitation education and ensure graduates are prepared for high-quality clinical care.

## CRediT authorship contribution statement

**Kheizaran Miri:** Writing – review & editing, Writing – original draft, Supervision, Data curation. **Alireza Sardashti:** Visualization, Investigation, Funding acquisition, Formal analysis, Data curation. **Sajedeh Moradi:** Supervision, Project administration, Methodology, Data curation, Conceptualization. **Arefeh Naseri:** Writing – review & editing, Writing – original draft, Investigation. **Amirhossein Mirzaei:** Writing – review & editing, Writing – original draft, Methodology, Data curation.

## Funding

This study was financially supported by the Research Deputy of Torbat Heydariyeh University of Medical Sciences.

## Declaration of competing interest

The authors declare that they have no known competing financial interests or personal relationships that could have appeared to influence the work reported in this paper.
