# Supplementary material for: Identifying clinical error patterns in nursing students’ CPR performance: a mixed-methods OSCE study
Source: Resusc Plus. 2025 Sep 5;26:101089. doi: 10.1016/j.resplu.2025.101089 (PMC12466282; doi:10.1016/j.resplu.2025.101089)
Supplement: Supplementary Data 1 [file mmc1.docx]

**Supplementary File 1: GRASPS Checklist Compliance**

This document demonstrates how our study adheres to the GRASPS (Guidelines for Reporting Simulation-Based Research in Healthcare Education) reporting standards. Below is the detailed checklist:

| **GRASPS Item** | **Where Addressed in Study** | **Page/ Section** | **Description of Compliance** |
| --- | --- | --- | --- |
| **1. Simulation Environment** | Methods: Phase One | Line 100 page 5 | Detailed description of Laerdal QCPR® smart manikin specifications (±1mm accuracy for depth, ±2/min for rate) and OSCE station setup (8-minute stations over 2 days). |
| **2. Participant Characteristics** | Results: Table 1 | Table files | Demographic data including age (21.8±0.7 years), gender distribution (57.9% male), and prior training (60.5% workshop attendance). |
| **3. Scenario Description** | Methods: Phase One | Line95 page 5 | Clear protocol for BLS/CPR station (14-station OSCE) with exclusion criteria (prior certification, incomplete participation). |
| **4. Simulation Fidelity Level** | Methods: Phase One | Line 103 page 5 | High-fidelity simulation using QCPR technology with real-time feedback capabilities aligned with AHA 2020 guidelines. |
| **5. Outcome Measures** | Methods: Phase One | Line 108 page 6 | Objective metrics: compression depth/rate, recoil, ventilation volume via QCPR software; subjective measures via interviews. |
| **6. Debriefing Process** | Methods: Qualitative Phase | Line 134 page 7 | Structured interviews (30-45 mins) with thematic analysis (MAXQDA 2022) and focus group validation (n=14). |
| **7. Limitations of Simulation** | Discussion: Limitations | Line 284 page 14 | Acknowledged artificial manikin use vs. real-patient variability and single-center design. |

**Key Explanatory Notes for the GRASPS Checklist**

1. **Simulation Environment (Item 1):**
   - The Laerdal QCPR® system provided **high-precision, continuous data sampling** (±1mm for depth), meeting GRASPS requirements for technical transparency.
   - OSCE timing (8 mins/station) and structure (14 stations) were explicitly described to ensure reproducibility.
2. **Participant Characteristics (Item 2):**
   - Table 1 included **critical covariates** (gender, training history) that may affect performance, as recommended by GRASPS.
3. **Scenario Description (Item 3):**
   - Standardized exclusion criteria (e.g., prior CPR certification) minimized confounding variables, aligning with GRASPS’ emphasis on scenario clarity.
4. **Outcome Measures (Item 5):**
   - Combined **quantitative** (QCPR metrics) and **qualitative** (interview themes) data addressed GRASPS’ call for comprehensive evaluation.
5. **Debriefing (Item 6):**
   - Thematic analysis of interviews and focus group validation followed GRASPS’ standards for **rigorous qualitative reporting**.
6. **Limitations (Item 7):**
   - Transparent acknowledgment of manikin limitations (e.g., lack of real-patient variability) fulfills GRASPS’ ethical reporting requirements.
