# Supplementary material for: Identifying clinical error patterns in nursing students’ CPR performance: a mixed-methods OSCE study
Source: Resusc Plus. 2025 Sep 5;26:101089. doi: 10.1016/j.resplu.2025.101089 (PMC12466282; doi:10.1016/j.resplu.2025.101089)
Supplement: Supplementary Data 2 [file mmc2.docx]

**Supplementary 1:**

**Checklist for Analysis of Clinical Errors and Success Rates in BLS Skills**

| **Skill Category** | **Specific Step in OSCE** | **Standard Guideline** | **Error Frequency (%)** | **Success Rate (%)** |
| --- | --- | --- | --- | --- |
| **Clinical Decision-Making** | Assesses the scene for safety | AHA Safety Check |  |  |
|  | Checks responsiveness (tap shoulders, call out) | AHA Responsiveness Check |  |  |
|  | Checks breathing (5-10 sec) | AHA Breathing Assessment |  |  |
|  | Checks pulse (5-10 sec) | AHA Pulse Check |  |  |
|  | Determines outcome post-2min CPR | AHA Post-CPR Evaluation |  |  |
|  | Places patient in recovery position | AHA Recovery Position |  |  |
|  | Calls emergency unit (115) | AHA Emergency Activation |  |  |
| **Chest Compressions** | Exposes the victim’s chest | AHA Chest Exposure |  |  |
|  | Positions hands correctly | AHA Hand Placement |  |  |
|  | Ensures proper hand angle | AHA Hand Angle |  |  |
|  | Performs compressions with correct depth (50-60 mm) | 50-60 mm depth (AHA) |  |  |
|  | Performs compressions at correct rate (100-120/min) | 100-120 compressions/min (AHA) |  |  |
|  | Allows full chest recoil | 100% recoil (AHA) |  |  |
| **Artificial Ventilation** | Establishes open airway (Head Tilt/Chin Lift or Jaw Thrust) | AHA Airway Management |  |  |
|  | Delivers two rescue breaths (500-600 mL) | AHA Rescue Breaths |  |  |
|  | Monitors chest rise | AHA Ventilation Monitoring |  |  |
|  | Maintains 30:2 compression-to-breath ratio | AHA CPR Ratio |  |  |
|  | Ensures proper ventilation | AHA Ventilation Effectiveness |  |  |
| **AED Operation** | Recognizes an AED | AHA AED Recognition |  |  |
|  | Uses AED correctly (pad placement, charge, shock delivery) | AHA AED Application |  |  |
